# Substituting abacavir for hyperlipidemia-associated protease inhibitors in HAART regimens improves fasting lipid profiles, maintains virologic suppression, and simplifies treatment

**DOI:** 10.1186/1471-2334-5-2

**Published:** 2005-01-12

**Authors:** Philip H Keiser, Michael G Sension, Edwin DeJesus, Allan Rodriguez, Jeffrey F Olliffe, Vanessa C Williams, John H Wakeford, Jerry W Snidow, Anne D Shachoy-Clark, Julie W Fleming, Gary E Pakes, Jaime E Hernandez, for the ESS40003 Study Team

**Affiliations:** 1 University of Texas Southwestern Medical Center, Dallas, Texas, USA; 2 North Broward Hospital, Ft. Lauderdale, Florida, USA; 3 IDC Research Initiative, Altamonte Springs, Florida, USA; 4 University of Miami, Miami, Florida, USA; 5 Swedish Hospital, Seattle, Washington, USA; 6 GlaxoSmithKline, Research Triangle Park, North Carolina, USA

## Abstract

**Background:**

Hyperlipidemia secondary to protease inhibitors (PI) may abate by switching to anti-HIV medications without lipid effects.

**Method:**

An open-label, randomized pilot study compared changes in fasting lipids and HIV-1 RNA in 104 HIV-infected adults with PI-associated hyperlipidemia (fasting serum total cholesterol >200 mg/dL) who were randomized either to a regimen in which their PI was replaced by abacavir 300 mg twice daily (n = 52) or a regimen in which their PI was continued (n = 52) for 28 weeks. All patients had undetectable viral loads (HIV-1 RNA <50 copies/mL) at baseline and were naïve to abacavir and non-nucleoside reverse transcriptase inhibitors.

**Results:**

At baseline, the mean total cholesterol was 243 mg/dL, low density lipoprotein (LDL)-cholesterol 149 mg/dL, high density lipoprotein (HDL)-cholesterol 41 mg/dL, and triglycerides 310 mg/dL. Mean CD4+ cell counts were 551 and 531 cells/mm^3 ^in the abacavir-switch and PI-continuation arms, respectively. At week 28, the abacavir-switch arm had significantly greater least square mean reduction from baseline in total cholesterol (-42 vs -10 mg/dL, *P *< 0.001), LDL-cholesterol (-14 vs +5 mg/dL, *P *= 0.016), and triglycerides (-134 vs -36 mg/dL, *P *= 0.019) than the PI-continuation arm, with no differences in HDL-cholesterol (+0.2 vs +1.3 mg/dL, *P *= 0.583). A higher proportion of patients in the abacavir-switch arm had decreases in protocol-defined total cholesterol and triglyceride toxicity grades, whereas a smaller proportion had increases in these toxicity grades. At week 28, an intent-to treat: missing = failure analysis showed that the abacavir-switch and PI-continuation arms did not differ significantly with respect to proportion of patients maintaining HIV-1 RNA <400 or <50 copies/mL or adjusted mean change from baseline in CD4+ cell count. Two possible abacavir-related hypersensitivity reactions were reported. No significant changes in glucose, insulin, insulin resistance, C-peptide, or waist-to-hip ratios were observed in either treatment arm, nor were differences in these parameters noted between treatments.

**Conclusion:**

In hyperlipidemic, antiretroviral-experienced patients with HIV-1 RNA levels <50 copies/mL and CD4+ cell counts >500 cells/mm^3^, substituting abacavir for hyperlipidemia-associated PIs in combination antiretroviral regimens improves lipid profiles and maintains virologic suppression over a 28-week period, and it simplifies treatment.

## Background

Protease inhibitors (PIs) used as components of highly active antiretroviral therapy (HAART) have been well documented to reduce both the morbidity and mortality associated with HIV infection [1,2]. However, many PI-based HAART regimens incur treatment-limiting side effects, interactions with concomitant medications, high daily pill burdens, and dietary and/or fluid requirements, making adherence to treatment a challenge [3]. Even more problematic from a long-term treatment perspective are the metabolic adverse effects, such as hyperlipidemia, insulin resistance, and lipodystrophy, which along with HIV infection itself, may constitute major risk factors for the development of coronary artery disease (CAD) [4].

The incidence of hyperlipidemia varies among PIs. The Swiss HIV Cohort Study showed that over 470 days, HIV-infected patients experienced a mean increase in total cholesterol of 77 mg/dL with ritonavir (*n *= 46), 46 mg/dL with nelfinavir (*n *= 21), 31 mg/dL with indinavir (*n *= 26), and 4 mg/dL with non-PI-containing antiretroviral regimens (*n *= 28) [5]. At the end of the study, total cholesterol exceeded 240 mg/dL in 44% of the ritonavir group, 35% of the indinavir group, 33% of the nelfinavir group, and 14% of the non-PI group. Other research has shown that some of the more recently developed PIs, including fosamprenavir and atazanavir, have a low likelihood of causing significant lipid elevation or adversely affecting the total cholesterol: high density lipoprotein (HDL) cholesterol ratio [6-8]. Although HIV infection itself is associated with above-normal triglyceride levels due to disease-related reduction in triglyceride clearance and increase in de-novo hepatic lipogenesis [9], some PIs exacerbate this lipid elevation by down-regulating low density lipoprotein (LDL)-receptor expression [10], interfering with the proteasome-mediated degradation of activated nuclear sterol regulatory element-binding protein-1 [11], and reducing triglyceride clearance further [12].

Because PIs have been available for less than a decade, it may be too early to confirm an association between PI usage and a more rapid onset of CAD. Epidemiological studies to date have reported varying findings regarding a PI-CAD link [13-15]. Until questions about such a possible link are resolved, it appears prudent to treat HIV-infected patients who have CAD risk factors with combinations of antiretroviral agents that produce maximal virologic suppression with the fewest lipid-elevating effects and metabolic adverse events.

Unlike most PIs, the nucleoside reverse transcriptase inhibitor (NRTI) abacavir is administered as just one tablet twice daily with no requirements regarding extra hydration or dosing with or without meals [16]. Abacavir is unlikely to be involved in drug interactions because it does not affect CYP3A4 drug metabolism, its use does not affect lipids or metabolic parameters, and it does not cause lipodystrophy [16]. These features of abacavir have prompted its use in substituting for components of HAART regimens in order to simplify treatment and to obviate the risk of drug interactions, fat maldistribution, and metabolic complications [17]. HAART regimens containing abacavir have been shown in direct comparative trials in HIV-infected patients with a baseline viral load <100,000 copies/mL to be as effective as nelfinavir-and indinavir-containing HAART in suppressing viral load and increasing CD4+ cell counts without inducing hyperlipidemia, insulin resistance, or central adiposity [18-21]. In patients with a baseline viral load ≥ 100,000 copies/mL, abacavir in combination with lamivudine and zidovudine has been shown to suppress viral load and increase CD4+ cell counts as well as nelfinavir-containing HAART [20], and was as effective as indinavir-containing HAART in one trial [18] but not in another [21]. In view of these findings, we conducted a 28-week, open-label pilot study (ESS40003) to compare the changes in PI-associated hyperlipidemia (fasting serum total cholesterol >200 mg/dL) in treatment-experienced HIV-infected patients with HIV-1 RNA <50 copies/mL who either substituted abacavir for the PI component in their regimen or continued the same PI-containing regimen.

## Methods

### Patient selection

Male and non-pregnant, non-lactating female outpatients were eligible for study enrollment if they were at least 18 years of age; had HIV-1 infection (documented by HIV-1 antibody enzyme-linked immunosorbent assay and confirmed by Western blot test of HIV-1 antibody, or positive HIV-1 blood culture, positive HIV serum antigen, or plasma viremia); had fasting serum total cholesterol >200 mg/dL; were stabilized on a well-tolerated PI-containing antiretroviral regimen for >3 months (either 2 NRTIs + 1 PI, or 2 NRTIs + 2 PIs if one PI was ritonavir); were naïve to abacavir and all non-nucleoside reverse transcriptase inhibitors (NNRTIs); and if their two most recently reported consecutive plasma HIV-1 RNA values were <400 copies/mL prior to screening and HIV-1 RNA was <50 copies/mL at screening. CD4+ cell counts could be of any magnitude. Patients were also eligible if their HAART regimen had changed due to intolerance (one drug substitution, such as a PI for another PI and/or an NRTI for another NRTI) >12 weeks prior to study start; or if their initial HAART regimen consisted of 2 NRTIs followed within 1 year by the addition of a PI. Patients were excluded from the study if they had genetically-related lipid disorders (familial lipoprotein lipase deficiency, apoprotein CII deficiency, type 3 hyperlipoproteinemia, hypercholesterolemia, hypertriglyceridemia, or combined hyperlipidemia); took a hypolipidemic or antidiabetic drug within 30 days of screening; had an AIDS-defining opportunistic infection or disease within 30 days of study entry; had a history of angina, anginal symptoms, and/or myocardial infarction; were substance abusers; or had a malabsorption syndrome that could interfere with absorption of the study medications. Patients provided written informed consent to participate in the study.

### Study design and treatment

This Phase IV, parallel group, active control, randomized, open-label, multicenter trial was conducted between November 1999 and November 2001 at 44 outpatient sites in the United States. An Institutional Review Board approved the study protocol at each site. To determine study eligibility, study candidates underwent a medical history, physical examination, CDC classification, clinical chemistry, hematology, and β-human chorionic gonadotropin test (women of childbearing age only) at the screening visit within 14 days pre-study. Patients meeting entry criteria at screening were randomized to either continued therapy with their current PI-containing antiretroviral regimen or to the same regimen with abacavir (300 mg twice daily) substituted for the PI(s). Abacavir was supplied as 300-mg tablets of Ziagen^® ^(GlaxoSmithKline, Research Triangle Park, North Carolina). In the abacavir-switch arm, patients received both abacavir and the hyperlipidemia-associated PI with their usual two-NRTI background combination for the first 4 weeks, after which the PI component of the regimen was discontinued. Co-administration of abacavir and the PI was done during the first 4 weeks to make sure that there was virologic coverage in case a patient developed a suspected abacavir-related hypersensitivity reaction, which would have necessitated stopping abacavir. Blood was sampled for lipid, viral load, and laboratory value measurements from fasted patients at baseline, and at weeks 4, 8, 12, 20, and 28. Patients who experienced HIV-1 RNA breakthrough (defined as HIV-1 RNA values between 50 and 1000 copies/mL by Roche Amplicor Ultrasensitive Assay) during the study period could receive intensification with efavirenz 600 mg once daily.

### Assessment of lipids

A complete fasting lipid panel was obtained from blood samples at baseline and at weeks 4, 8, 12, 20, and 28 to allow measurement of the primary study endpoint (change from baseline in fasting serum total cholesterol) and secondary study endpoints (change from baseline in LDL-cholesterol, very low density lipoprotein (VLDL)-cholesterol, high density lipoprotein (HDL)-cholesterol, and triglycerides). Apolipoproteins B and E, free fatty acids, and LDL subfractions were measured at baseline and week 28. Direct LDL-cholesterol was measured in all patients at baseline, week 4, and week 28, as well as any treatment visit at which time a fasting triglyceride >400 mg/dL was observed.

Fasting serum total cholesterol and triglyceride levels were measured enzymatically by using cholesterol/HP reagent (Roche Diagnostics, Indianapolis, Indiana) and triglyceride reagent (GPO-Trender) (Roche Diagnostics, Ibid). HDL-cholesterol was measured enzymatically in the supernatant formed following centrifugation of serum mixed with a polyanion dextran sulfate/divalent magnesium solution (Roche Diagnostics, Ibid). LDL- and VLDL-cholesterol were estimated by the Friedewald equation [22]. Direct LDL-cholesterol was measured by a nonionic detergent method using alpha-cyclodextrin/4-aminoantipyrin (Roche Diagnostics, Ibid.). Apolipoprotein B was measured using the apolipoprotein B antigen-antibody reaction method employing the Beckman IMMAGE Immunochemistry System (Beckman Instrument, Brea, California), and apolipoprotein E by a phosphate buffer/anti-human apolipoprotein E method employing a Wako Apolipoprotein Calibrator (WAKO Chemicals, Richmond, Virginia). LDL subfractions were measured by electrophoresis (Pacific Biometrics, Seattle, Washington) [23], and non-esterified free fatty acids were measured by the WAKO enzymatic colorimetic method (WAKO Chemicals, Ibid.).

Four protocol-defined toxicity grades for hyperlipidemia were assigned during the study for serum cholesterol (Grade 1 for values >1–1.3 times the upper limit of normal [ULN], Grade 2 for >1.3–1.6 times the ULN, Grade 3 for >1.6–2 times the ULN; and Grade 4 for >2 times the ULN) and serum triglycerides (Grade 1 for ULN-399 mg/dL, Grade 2 for 400–750 mg/dL, Grade 3 for 751–1200 mg/dL, and Grade 4 for >1200 mg/dL).

### Efficacy assessment

Changes in HIV-1 RNA levels and CD4+ cell counts were secondary endpoints in this study. Plasma HIV-1 RNA levels were measured in blood samples at screening and at all study visits using both the Roche AMPLICOR PCR Standard 1.0 assay (lower limit of quantitation [LLOQ] 400 copies/mL) and the Roche PCR assay Amplicor HIV-1 MONITOR UltraSensitive Version 1.0 (LLOQ 50 copies/mL) (both assays from Roche Diagnostics, Branchburg, New Jersey). Virologic failure was defined as HIV-1 RNA >1000 copies/mL on two occasions at least 1 week apart. CD4+ cell counts were determined by flow cytometry at baseline, and at weeks 4, 12, 20, and 28.

### Safety assessment

Frequency and severity of all clinical and laboratory adverse experiences were assessed at each visit. A cardiovascular disease risk factor assessment was conducted at baseline only. Body mass index (BMI) and waist-to-hip ratio (WHR) were measured at baseline and at weeks 4, 8, 12, 20 and 28. Three types of waist measurements were conducted: mid, minimal, and umbilicus. Insulin resistance measures (C-peptide and fasting insulin to glucose ratio) were assessed at baseline and at weeks 4, 8, 12, 20, and 28. Leptin and lactate were measured at baseline and week 28.

Diagnosis of possible abacavir-related hypersensitivity reaction was to be considered if the following multi-organ signs and symptoms appeared in a patient following initiation of abacavir: fever, rash, gastrointestinal symptoms (nausea, vomiting, diarrhea, or abdominal pain), lethargy, or malaise with or without concomitant respiratory symptoms (dyspnea, sore throat, cough), musculoskeletal symptoms (myalgia, myolysis, arthralgia), headache, paresthesia, and edema. No rechallenge of abacavir was permitted in patients developing this syndrome. The definition and usual clinical presentation of abacavir-related hypersensitivity has been defined previously [24,25].

### Adherence and health outcomes assessments

Adherence was assessed by the Patient Medication Adherence Questionnaire-7 (PMAQ-7) [26]. The PMAQ-7 is a self-reported measure of adherence that patients completed at baseline and at weeks 4, 8, 12, 20, and 28 (or upon permanent discontinuation due to virologic failure or toxicity). In the PMAQ-7, patients were asked to indicate the number of doses of each medication they took during each of the previous 7 days. An overall regimen adherence rate was calculated at each week as the proportion of doses actually taken relative to the number of doses prescribed summed across each medication within the regimen. The PMAQ-7 also measured barriers and motivators to adherence.

Health-related quality of life (QOL) was evaluated using the Medical Outcomes Study 36-item Short Form Health Survey (SF-36) [27], which patients completed at baseline and at study weeks 12 and 28. Healthcare resource utilization (total health care visits, emergency room visits, intensive care hospitalizations [nights], general ward hospitalizations [nights], outpatient clinic visits, home care visits, and long-term care visits [nights]) was assessed at weeks 4, 8, 12, 20 and 28.

### Statistical analysis

A sample size of 80 patients per treatment arm was deemed necessary for 80% power to detect a difference of 20 mg/dL in fasting total cholesterol between groups using a two-group *t*-test with a two-sided significance level of 0.05, assuming a dropout rate of 20%. This sample size calculation is based on data from NZTA4002, in which the standard deviation of total cholesterol in the nelfinavir/zidovudine/lamivudine group at baseline was 39.5 mg/dL.

The primary efficacy population was the intent-to-treat (ITT) population, which consisted of all patients who were randomized into the study. The safety population consisted of all randomized patients who received at least one dose of study drug. Analysis of covariance (ANCOVA) was used to compare the least squares means (LSM) of the two treatment groups. The LSM represented the mean value adjusted for the average value of the covariate from both treatment groups. LSMs of change from baseline in serum lipids (total, LDL, VLDL, and HDL-cholesterol, LDL subfractions, triglycerides, free fatty acids), apolipoprotein B, apolipoprotein E, leptin, C-peptide, glucose, insulin, and lactate were reported. The ANCOVA model included terms for treatment group, baseline value of the variable of interest, PI strata and gender.

Two types of analyses were performed with HIV-1 RNA data: the ITT: observed analysis and the ITT missing equals failure analysis (ITT: M = F). In the ITT: observed analysis, only available assessments were used (no imputation for missing values), regardless of whether the patient was still receiving their original therapy. In the ITT: M = F analysis, all missing values were considered as failure. Proportions of patients with HIV-1 RNA <400 copies/mL and <50 copies/mL were compared between treatment groups with a 95% confidence interval (CI) on the difference between proportions.

Differences in domain scores to the SF-36 and the PMAQ-7 were compared using the Wilcoxon rank sum test. Differences between treatment arms in the incidence of treatment-related adverse events by body system were evaluated by Fisher's Exact test. A *P *value of 0.05 was considered statistically significant.

## Results

### Patient characteristics and disposition

**Figure 1:**
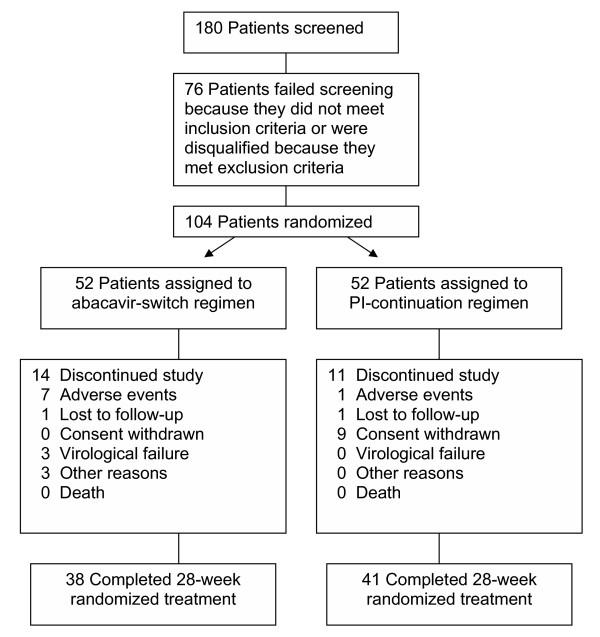
Profile of patient enrollment and discontinuations through 28 weeks of treatment.

Of 104 patients enrolled in the study, 52 were randomized to the abacavir-switch arm and 52 to the PI-continuation arm (Figure 1). Most of the patients were males (89%), and the median age was 42 years (Table 1). The study population was ethnically diverse; approximately one-half were Caucasian, one-quarter African American, and one-quarter Hispanic. Mean CD4+ cell counts were 551 and 531 cells/mm^3 ^in the abacavir-switch and PI-continuation arms, respectively. About two-thirds of the patients were HIV Category A. The abacavir-switch and PI-continuation arms were well matched with respect to mean baseline HIV-1 RNA levels (1.727 vs 1.699 log_10 _copies/mL, *P *= 0.062), total cholesterol (244 vs 241 mg/dL), LDL-cholesterol (149 vs 149 mg/dL), VLDL-cholesterol (68 vs 56 mg/dL), HDL-cholesterol (39 vs 42 mg/dL), triglycerides (340 vs 280 mg/dL), CAD risk factors (Table 1), and specific PI used immediately pre-study (>80% receiving either nelfinavir or indinavir). There were no significant differences between treatment arms with respect to baseline BMI, waist-to-hip ratios or insulin resistance. During treatment, the background NRTIs used in the abacavir-switch and PI-continuation arms were similar and included most commonly the lamivudine 150 mg/zidovudine 300 mg combination tablet (Combivir^® ^[GlaxoSmithKline, Ibid.], 52% vs 47%), stavudine (36% vs 42%), lamivudine (32% vs 40%), and zidovudine (6% vs 2%).

**Table 1:** Characteristics and disposition of the study patients (intent-to-treat population)

Characteristic	Abacavir-switch arm (N = 52)	PI-continuation arm (N = 52)	Total study population (N = 104)
Age, y			
Median (range)	43 (23–64)	42 (25–62)	42 (23–64)
Sex, No. (%)			
Male	46 (88)	47 (90)	93 (89)
Female	6 (12)	5 (10)	11 (11)
Race, No. (%)			
Caucasian	26 (50)	28 (54)	54 (52)
African American	16 (31)	11 (21)	27 (26)
Hispanic	10 (19)	13 (25)	23 (22)
Mean HIV-1 RNA, log_10 _copies/mL (SD)	1.73 (0.16)	1.69 (0.04)	1.71 (0.06)
Mean CD4+ cell count, cells/mm^3 ^(SD)	551 (226)	531 (233)	541 (229)
Mean weight, kg (SD)	79.3 (16.8)	80.4 (16.6)	79.8 (16.7)
Mean BMI, kg/cm^2 ^(SD)	25.9 (4.5)	26.4 (4.9)	26.1 (4.7)
Mean waist-to-hip ratio	0.94 (0.06)	0.93 (0.07)	0.94 (0.06)
CDC Class, n (%)			
Category A	33 (63)	34 (65)	67 (64)
Category B	10 (19)	11 (21)	21 (20)
Category C	9 (17)	7 (13)	16 (15)
Mean (SD) total cholesterol, mg/dL	244 (45)	241 (44)	
Mean (SD) LDL cholesterol, mg/dL	149 (34)	149 (30)	
Mean (SD) HDL cholesterol, mg/dL	39 (15)	42 (14)	
Mean (SD) triglycerides, mg/dL	340 (213)	280 (282)	
Coronary artery disease risk factors, n (%)*	34 (65)	26 (50)	60 (58)
Age	18 (35)	12 (23)	30 (29)
Family history	4 (8)	4 (8)	8 (8)
Cigarette smoking	19 (37)	13 (25)	32 (31)
Hypertension	5 (10)	8 (15)	13 (13)
Low HDL	10 (19)	4 (8)	14 (13)
Diabetes mellitus	3 (6)	0	3 (3)
Antiretroviral medications taken prior to screening, n (%)			
Any	28 (56)	20 (44)	48 (46)
NRTIs			
Zidovudine	16 (32)	9 (20)	25 (24)
Lamivudine	12 (24)	8 (18)	20 (19)
Stavudine	5 (10)	2 (4)	7 (7)
Didanosine	7 (14)	0	7 (7)
Zalcitabine	3 (6)	0	3 (3)
NNRTIs			
Efavirenz	1 (2)	0	
PIs			
Indinavir	11 (22)	8 (18)	19 (18)
Nelfinavir	4 (8)	2 (4)	6 (6)
Ritonavir	2 (4)	1 (2)	3 (3)
Saquinavir	2 (4)	0	2 (2)
PI used at screening			
Nelfinavir	22 (42)	21 (40)	43 (41)
Indinavir	21 (40)	22 (42)	43 (41)
Saquinavir SGC	6 (12)	6 (12)	12 (12)
Amprenavir	2 (4)	2 (4)	4 (4)
Ritonavir	1 (2)	1 (2)	2 (2)
Premature withdrawal from study, n (%)	14 (27)	11 (21)	25 (24)
Adverse event	7 (13)	1 (2)	8 (8)
Consent withdrawn	0	9 (17)	9 (9)
Lost to follow-up	1 (2)	1 (2)	2 (2)
Protocol violation	2 (4)	0	2 (2)
Protocol-defined virologic failure	3 (6)	0	3 (3)
Other	1 (2)	0	1 (1)

*Cumulative coronary artery disease risk factors in the abacavir-switch arm: 0 factors = 18; 1 factor = 16; 2 factors = 12; 3 factors = 5; 4 factors = 1. Cumulative coronary artery risk factors in the PI-continuation arm: 0 factors = 26; 1 factor = 12; 2 factors = 13; and 3 factors = 1.

*Abbreviations: *AIDS = acquired immune deficiency syndrome; ART = antiretroviral therapy; CDC = Centers for Disease Control; HDL = high density lipoprotein; HIV-1 = human immunodeficiency virus type 1; LDL = low density lipoprotein; LSM = least squares mean; SD = standard deviation; SGC = soft gel capsule.

A similar number of patients withdrew prematurely from the study for the reasons delineated in Table 1. Comparatively more patients withdrew due to adverse events in the abacavir-switch arm (7/52 vs 1/52) and due to consent withdrawn in the PI-continuation arm (0 vs 9/52 [all withdrawals occurred after the patients learned they were not receiving abacavir]). The difference between the two treatment arms regarding number of premature withdrawals due to the sum of adverse events plus virologic failure was not statistically significant (*P *= 0.144). No patient required efavirenz intensification of their treatment regimen.

### Changes in lipids

**Figure 2:**
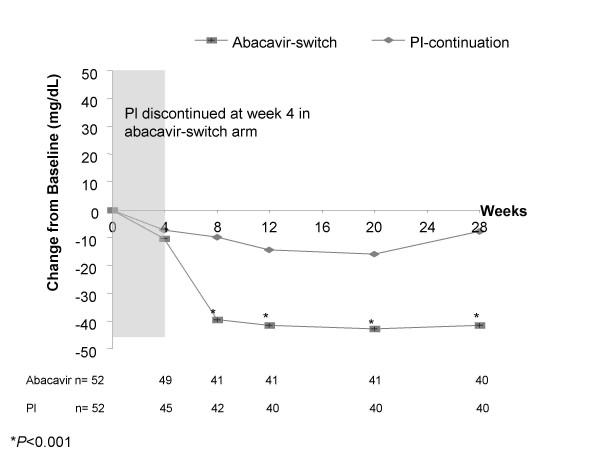
Least squares mean change from baseline in fasting serum total cholesterol.

**Figure 3:**
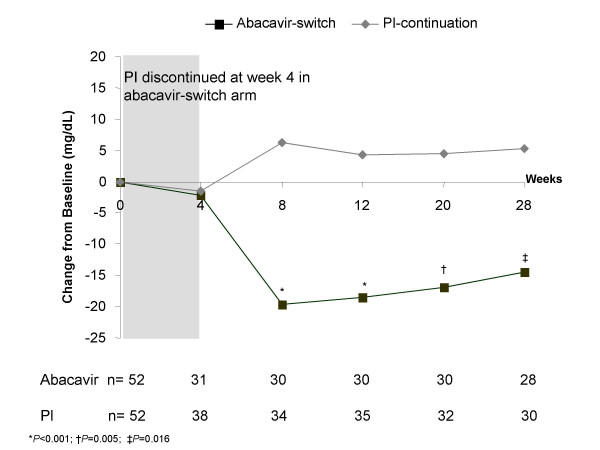
Least squares mean change from baseline in fasting serum LDL-cholesterol.

In the ITT analysis, the abacavir-switch arm experienced greater reductions in total cholesterol (Figure 2) and LDL-cholesterol (Figure 3) than the PI-continuation arm over the entire study period, with differences between treatment arms being statistically significant from week 8 onward. At week 28, patients in the abacavir-switch arm had a significantly greater LSM decrease from baseline in total cholesterol (-42 vs -10 mg/dL; *P *<0.001), LDL-cholesterol (-14 vs +5 mg/dL; *P *= 0.016), LDL direct-cholesterol (-15 vs +1 mg/dL; *P *= 0.028), VLDL-cholesterol (-27 vs -7 mg/dL; *P *= 0.019), triglycerides (-134 vs -36 mg/dL; *P *= 0.019), apolipoprotein B (-23 vs -11 mg/dL; *P *= 0.031), and apolipoprotein E (-2 vs -1 mg/dL; *P *= 0.021) versus the PI-continuation arm. Over the study period, no significant differences were observed between the abacavir-switch or PI-continuation arms with respect to LDL subfractions (particle size A and major class) or HDL-cholesterol (LSM change from baseline at week 28, +0.2 vs +1.3 mg/dL, *P *= 0.583; LSM at week 28, 40 vs 41 mg/dL). The abacavir-switch arm showed a trend for greater LSM reduction from baseline in free fatty acids (-0.3 vs -0.2 mEq/L; *P *= 0.052).

**Figure 4:**
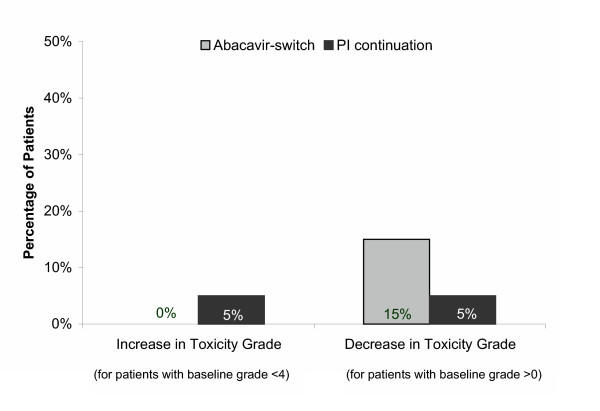
Changes in cholesterol toxicity grade.

The LSM value for total cholesterol was below the National Cholesterol Education Program Adult Treatment Panel III (NCEP ATP III) goal value (<200 mg/dL) in the abacavir-switch arm (198 mg/dL); however, it remained above this value in the PI-continuation arm (230 mg/dL). The LSM LDL-cholesterol level at week 28 was 133 mg/dL in the abacavir-switch arm and 153 mg/dL in the PI-continuation arm (*P *= 0.016). A higher proportion of patients in the abacavir-switch arm had shifts to lower toxicity grades as compared to the PI-continuation arm, whereas a lower proportion in the abacavir-switch arm had shifts from baseline in cholesterol to more severe toxicity grades (Figure 4)

**Figure 5:**
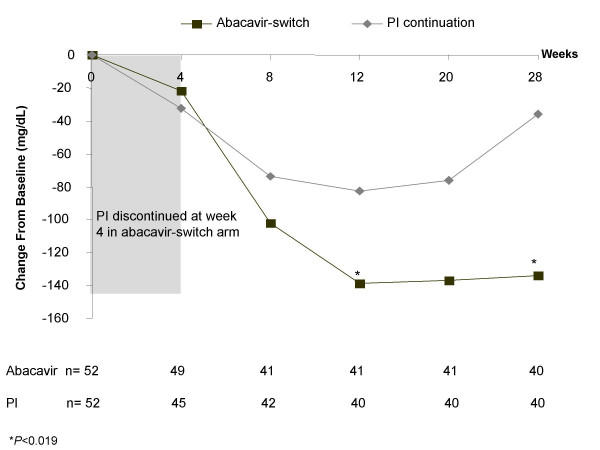
Least squares mean change from baseline in fasting serum triglycerides.

**Figure 6:**
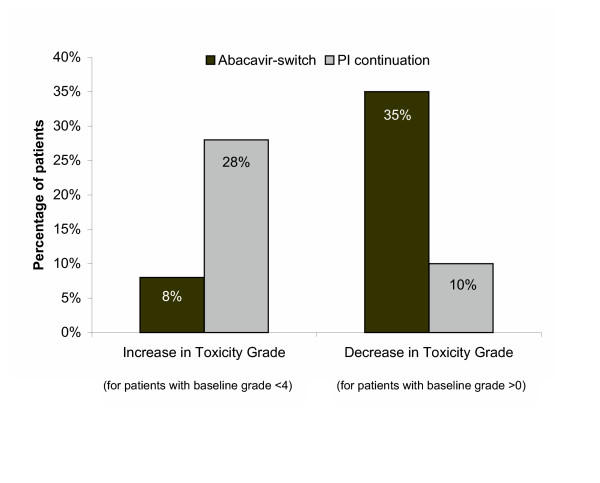
Changes in triglycerides toxicity grade.

Changes in serum triglycerides in the two treatment groups paralleled the changes in total cholesterol. At week 28, the LSM reduction in triglycerides was significantly greater in the abacavir-switch arm than the PI-continuation arm (-134 vs -36 mg/dL, *P *= 0.019) (Figure 5). A higher proportion of patients in the abacavir-switch arm had decreases in the triglyceride toxicity grade as compared to the PI-continuation arm, whereas a lower proportion in the abacavir-switch arm had increases in triglyceride toxicity grade (Figure 6).

### Efficacy

In the ITT: M = F analysis, no differences were observed between the abacavir-switch and PI-continuation treatment arms at any time during the study regarding proportions of patients achieving undetectable HIV-1 RNA. At week 28, HIV-1 RNA was <400 copies/mL in 69% [36/52] and 77% [40/52] of patients in the abacavir-switch and PI-continuation arms, respectively (*P *= 0.508; 95% CI [-0.25, 0.09]), and <50 copies/mL in 62% [32/52] and 75% [39/52] of patients in these respective groups (*P *= 0.206; 95% CI [-0.31, 0.04]).

The ITT: observed analysis showed that HIV-1 RNA remained <400 copies/mL in ≥ 90% of patients over the entire study duration. At week 28, the proportion of patients with HIV-1 RNA <400 copies/ml was 90% [36/40] and 100% [40/40] in the abacavir-switch and PI-continuation arms, respectively(*P *= 0.116; 95% CI [-0.19, -0.11]) (Table 2). A significantly higher proportion of patients in the PI-continuation arm achieved HIV-1 RNA levels <50 copies/ml at week 28 (98% [39/40] vs. 80% [32/40]; P = 0.029; 95% CI [-0.31, -0.04]).

**Table 2:** Proportion of patients with HIV-1 RNA <400 copies/mL and <50 copies/mL

Week	HIV-1 RNA <400 copies/mL, n/N (%)						HIV-1 RNA <50 copies/mL, n/N (%)				
	
	ITT: Observed			ITT: M = F			ITT: Observed			ITT: M = F	
	Abacavir	PI		Abacavir	PI		Abacavir	PI		Abacavir	PI
Baseline	52/52 (100)	52/52 (100)		52/52 (100)	52/52 (100)		42/52 (81)	47/52 (90)		42/52 (81)	47/52 (90)
4	49/49 (100)	44/45 (98)		49/52 (94)	44/52 (85)		48/49 (98)	43/45 (96)		48/52 (92)	43/52 (83)
8	39/42 (93)	42/42 (100)		39/52 (75)	42/52 (81)		38/42 (90)	40/42 (95)		38/52 (73)	40/52 (77)
12	38/41 (93)	40/40 (100)		38/52 (73)	40/52 (77)		38/41 (93)	39/40 (98)		38/52 (73)	39/52 (75)
20	38/41 (93)	40/40 (100)		38/52 (73)	40/52 (77)		32/41 (78)	38/40 (95)		32/52 (62)	38/52 (73)
28	36/40 (90)	40/40 (100)		36/52 (69)	40/52 (77)		32/40 (80)	39/40 (98)		32/52 (62)	39/52 (75)

*Abbreviations: *ITT = intent to treat; M = F = missing equals failure analysis; PI = protease inhibitor.

No differences were observed regarding time to virologic breakthrough (*P *= 0.961). Three patients (6%) in the abacavir-switch arm were protocol-defined virologic failures. However, one of these patients remained on a 2-NRTI/abacavir regimen after withdrawing from the study and his viral load declined. The second of these patients was noncompliant and discontinued his antiretroviral treatment prior to the confirmation blood draw for virologic failure. The third virologic failure in the abacavir-switch arm had a history of prior dual nucleoside therapy for >1 year.

At baseline, the mean CD4+ cell counts were 551 and 531 cells/mm^3 ^in the abacavir-switch and PI-continuation arms, respectively. Changes in CD4+ counts over the study period were similar. At week 28, the LSM CD4+ cell counts were 514 and 526 cells/mm^3^, respectively, with the LSM difference from baseline being -13 and -1 cells/mm^3^, respectively (*P *= 0.597).

### Adherence and health outcomes assessments

PMAQ-7 results showed no apparent differences between the abacavir-switch and PI-continuation arms in overall adherence at week 28 (90% vs 94%) or in the Social Support, Adaptability, Knowledge and Attitudes, and Memory and Recall domains. However, the abacavir-switch arm had significantly higher scores in the Scheduling and Timing domain for all study visits (*P *≤ 0.027 at each week) and in the Physical Effects domain for all visits (*P *< 0.05) except those at weeks 4 and 28. In answer to the PMAQ-7 questions "it is easy for me to take my medicines at the time I am supposed to" and "my medicines are convenient to take", more patients in the abacavir-switch arm answered "definitely true" at week 28 (51% vs 35% and 63% vs 29%, respectively). In the SF-36, the week 28 QOL scores did not differ between treatment arms for most domains, except Vitality (in favor of the PI-continuation arm; *P *= 0.042) and Health (in favor of the abacavir-switch arm; *P *= 0.013). There were no differences between treatment arms with respect to any health care utilization parameter.

### Safety

Substitution of PIs with abacavir in HAART regimens was generally well tolerated. No significant differences between treatment arms were observed for cardiovascular, endocrine/metabolic, hepatobiliary tract/pancreatic events, lower respiratory events, musculoskeletal, neurology, psychiatric, or skin adverse events, although more gastrointestinal (*P *= 0.002) and non-site specific adverse events (*P *= 0.003) were observed in the abacavir-switch arm. Treatment-related adverse events reported by >5% of patients in the abacavir-switch arm included nausea (12/50 [14%]), fatigue (6/50 [12%]), diarrhea (4/50 [8%]), and depressive disorders (3/50 [6%]). Two patients (4%) in the abacavir-switch arm experienced possible abacavir-related hypersensitivity reactions. Adverse events leading to treatment withdrawal in 7 patients in the abacavir-switch arm included possible abacavir-related hypersensitivity reaction (2), mild nausea (2), mild shortness of breath/tachycardia (1), mild disorientation (1), and combination of mild diarrhea, facial edema/numbness and malaise (1). Hyperlipidemia led to treatment withdrawal in 1 patient in the PI-continuation arm. No differences between the two treatments were observed in body weight, waist-to-hip ratio, BMI, lactate, leptin, glucose, insulin, or C-peptide.

## Discussion

The results of this study indicate that in hyperlipidemic, virologically suppressed, immunocompetent antiretroviral-experienced patients (HIV-1 RNA <50 copies/mL, CD4+ counts >500 cells/mm^3^), substituting abacavir for hyperlipidemia-associated PIs in HAART regimens improves lipid profiles and maintains virologic suppression over a 28-week period. The lipid findings are consistent with those of other studies in which abacavir was substituted for PIs in HAART regimens [28-33]. However, unlike the other studies, ESS40003 evaluated a population consisting entirely of patients who were hyperlipidemic at baseline. Also, unlike two previous studies that reported lipid changes following abacavir substitution [29,30], ESS40003 measured *fasting *lipids rather than lipids in a non-fasted state (which may confound results). Thus, true differences between abacavir and PI-containing regimens could be determined, and lipid criteria established by the NCEP ATP III could be applied [34]. In addition to monitoring changes in cholesterol and triglycerides, ESS40003 measured changes in other laboratory values known to contribute to atherogenesis (apolipoproteins B and E, LDL subfractions, and indicators of changes in lipid processing [leptin and free fatty acids]) to gain a better understanding of the extent of the improvement in lipid profile following the switch to abacavir. Substitution of hyperlipidemia-associated PIs with abacavir was expected to improve lipid profiles because results of direct comparisons of abacavir/NRTI regimens with PI/NRTI regimens (same NRTIs administered) in antiretroviral-naïve patients have shown no significant effects on lipids with abacavir/NRTI-containing regimens [18-20].

Cross-trial comparisons with other abacavir-switch studies regarding lipid changes are limited by differences between studies in prevalence and severity of hyperlipidemia prior to the switch, specific PIs administered, pre-existing CAD risk factors of the particular patients enrolled, and stipulated dietary and exercise restrictions. Nevertheless, some generalizations can be made between the lipid changes noted in ESS40003 and those reported in other abacavir-switch studies [28-33]. First, as in these other studies, decreases in total cholesterol, LDL-cholesterol, and triglycerides occurred rapidly following the switch to abacavir, with statistically significant differences noted between treatment arms within 4 weeks. Second, as would be expected, the magnitude of the reduction in total- and LDL-cholesterol and triglycerides at week 28 in our study was markedly greater than that reported in studies that included normolipidemic as well as hyperlipidemic patients. Third, as in the other studies, switching from a PI to abacavir had no significant effect on HDL-cholesterol.

The small reduction in lipids observed in the PI-continuation arm may have occurred because patients were aware that their lipids were being monitored, and therefore may have exerted more self-control than usual regarding their dietary fat intake and frequency of smoking, or may have exercised more (not monitored in this study). A similar phenomenon was observed over 48 weeks in another PI-to-abacavir switch study, CNA30017 [29]. As no change in body weight, BMI, or waist-to-hip ratio was observed in our study in either group, anthropomorphic parameters were unlikely to have accounted for the decreases in lipids observed during this study. The 28-week duration of this study may have been too short to see significant changes in BMI or waist-to-hip ratios. However, in the Swiss HIV Cohort Study, substitution of abacavir for a PI was not associated with a change in waist-to-hip ratios even after 48 weeks post-switch [30].

Maintenance of virologic suppression and increases in CD4+ cell count over the 24-week period following the switch to abacavir were expected in view of the lack of change in these surrogate markers previously observed in abacavir-switch studies conducted over 48 weeks to 1 year [27,29].

The abacavir-containing regimen was generally well-tolerated, and the type and incidence of adverse events (notably fatigue and nausea) and rare occurrence of suspected abacavir-related hypersensitivity reactions were consistent with what has been reported in other clinical trials [16]. New adverse events in the PI-continuation arm were not expected as patients had been stabilized on this treatment for >3 months. This fact may have accounted for the comparatively lower incidence of GI adverse events in the PI-continuation arm than in the abacavir-switch arm, a finding that has not been observed in direct comparisons of abacavir with PIs (indinavir or nelfinavir) administered with the same background antiretroviral drugs [18,19,21].

Results of the PMAQ-7 indicated significantly better Scheduling and Timing scores in the abacavir-switch arm. Significant improvement in this PMAQ-7 dimension was similarly reported at 24 weeks in the PI-to-abacavir switch study, COL30305 [33]. Improved scores in this dimension may have been related to the simpler, twice-daily dosing of abacavir compared to the patients' previous PI-containing regimens. The proportion of patients reporting 100% adherence previously was shown to be higher at 24 weeks in one PI-to-abacavir switch study (COL30305; 92% vs 68%) [33] and at 48 weeks in another (CNA30017; 91% vs 76%) [29]. In these trials, better adherence with the abacavir-containing regimen was believed to be due at least in part to the relatively lower pill count and absence of special dosing requirements incurred by abacavir-containing HAART. The absence of a significant difference in overall QOL between the abacavir-switch and PI-continuation arms was not unexpected in view of the same finding being demonstrated on the SF-36 at 24 weeks in COL30305 [33].

Switching to abacavir is just one of several switch strategies that have been investigated to date in an attempt to remedy hyperlipidemia in patients receiving PI-containing HAART. It is acknowledged that the ideal candidate for this switch strategy is a patient started on triple therapy, where pre-existing abacavir resistance is unlikely [30,32]. Another switch strategy-replacement of hyperlipidemia-associated PIs with PIs that have a low likelihood of causing significant lipid elevation or adversely affecting the total cholesterol:HDL cholesterol ratio (fosamprenavir or atazanavir)-would be expected to improve lipid profiles in HIV-infected patients [6-8,35]. Attempts to reduce lipids by switching from a hyperlipidemia-associated PI to NNRTIs have also been investigated [32,36-43]. More favorable lipid effects appear to occur when a switch is made to nevirapine than to efavirenz [42,43].

This study had several limitations. As patients in this study had mean CD4+ cell counts >500 cells/mm^3^, they were highly immunocompetent and may not be representative of typical HIV-infected patients presenting to their physician with PI-associated hyperlipidemia. The study did not evaluate the influence of NRTI background drugs on lipid changes because the patients remained on the same baseline NRTIs. This could have affected the results because stavudine is known to elevate cholesterol and triglyceride levels [44], whereas zidovudine and lamivudine do not. Most studies have found that switching therapy tends to be optimally effective in patients whose viral load is fully suppressed for at least 6 months rather than the 3 months in our study [45]. This shorter pre-study time of viral suppression could have biased the virologic results against abacavir, as could allowing prior suboptimal nucleoside therapy. As we did not have information about prior NRTI combinations that many patients received pre-study, we could not assess whether these earlier NRTI combination regimens had been inadequate. Lipid data for study participants prior to their receipt of PI-containing regimens were not available to the investigators; thus, whether PIs were the cause of the patients' hyperlipidemia could not be verified. Dietary intake and physical activity assessments were not performed in this study to evaluate whether either differed between the treatment arms.

Further valuable clinical information could have been acquired from this study had switch agents in addition to abacavir been used as comparators. In the only study of this type that has been conducted to date – the Nevirapine-Efavirenz-Abacavir (NEFA) Study – a significantly lower proportion of stabilized HAART recipients (HIV-1 RNA <50 copies/mL for = 6 months) switching from a PI to abacavir developed fasting plasma triglycerides >400 mg/dL and plasma cholesterol >240 mg/dL at 12 months compared to treatment groups in which patients switched from a PI to efavirenz or nevirapine [32]. Kaplan-Meier estimates of the likelihood of reaching the primary treatment end point (increase in HIV-1 RNA levels to ≥ 200 copies/mL, progression to AIDS, or death) in NEFA showed no significant differences between treatments. Median CD4+ cell counts increased above baseline similarly in all three treatment arms (by 39–50 cells/mm^3 ^at 12 months). However, the abacavir-switch arm had a significantly lower incidence of adverse events than the efavirenz-switch and nevirapine-switch arms (41% vs 57% and 54%, *P*= 0.03), a lower incidence of neuropsychiatric adverse events than the efavirenz group (9% vs 31%; Grade 3 or 4: 0.7% vs 14%), and significantly fewer cases of discontinuation of study drug due to adverse events (6% vs 17% and 17%, *P*= 0.01). Overall, NEFA, like our study, indicated that abacavir was an appropriate drug to substitute for PIs in HAART regimens as long as patients were virologically suppressed pre-switch and minimally antiretroviral-experienced. Our study differed from NEFA in that *all *participants in ESS40003 had PI-associated hyperlipidemia at baseline (NEFA included only 7–13% with triglycerides >400 mg/dL and 21–25% with total cholesterol >240 mg/dL) and because our study used a less stringent definition of virologic failure (HIV-1 RNA >1000 copies/mL on two occasions at least 1 week apart vs. HIV-1 RNA ≥ 200 copies/mL at ≥ 16 weeks, with subsequent confirmation [NEFA]).

## Conclusions

In conclusion, in antiretroviral-experienced patients with HIV-1 RNA <50 copies/mL and CD4+ counts >500 cells/mm^3^, substituting abacavir for hyperlipidemia-associated PIs in HAART regimens improves lipid profiles and maintains virologic suppression over a 28-week period, and it simplifies treatment.

## Competing interests

The author(s) declare that they have no competing interests.

## Authors' contributions

JEH, PHK and VCW conceived the study design, PHK, MGS, EDJ, AR, JFO, VCW, JWS reviewed and approved study design, VCW provided the statistical methods for the study and performed the statistical analysis of the results, EH, VCW, JHW, JWS wrote, reviewed and edited the protocol, GEP drafted manuscript and evaluated lipid data previously published in antiretroviral studies described in Background and Discussion, JWF, JEH, PHK, MGS, EDJ, AR, JFO, JEH, JWF, JHW, JWS, ADS-C, VCW reviewed and edited the manuscript, ADS-C, JWF, PHK, MGS, EDJ, AR, JFO enrolled study subjects, ADS-C, JWF, JEH, JHW monitored the study, ADS-C, JWF, JEH, JWS, VCW evaluated the clinical data from the study, ADS-C set up the study at study sites and JEH contributed to secure funding.

## Pre-publication history

The pre-publication history for this paper can be accessed here:


